# Necroptosis Takes Place in Human Immunodeficiency Virus Type-1 (HIV-1)-Infected CD4^+^ T Lymphocytes

**DOI:** 10.1371/journal.pone.0093944

**Published:** 2014-04-08

**Authors:** Ting Pan, Shuangxin Wu, Xin He, Haihua Luo, Yijun Zhang, Miaomiao Fan, Guannan Geng, Vivian Clarke Ruiz, Jim Zhang, Lisa Mills, Chuan Bai, Hui Zhang

**Affiliations:** 1 Institute of Human Virology, Sun Yatsen University, Guangzhou, China; 2 Key Laboratory of Tropical Disease Control of Ministry of Education, Zhongshan School of Medicine, Sun Yatsen University, Guangzhou, China; 3 Division of Infectious Diseases, Department of Medicine, Center for Human Virology, Thomas Jefferson University, Philadelphia, Pennsylvania, United States of America; Salute San Raffaele University School of Medicine, Italy

## Abstract

Human immunodeficiency virus type 1 (HIV-1) infection is characterized by progressive depletion of CD4^+^ T lymphocytes and dysfunction of the immune system. The numbers of CD4^+^ T lymphocytes in the human body are maintained constantly by homeostatic mechanisms that failed during HIV-1 infection, resulting in progressive loss of CD4^+^ T cells mainly via apoptosis. Recently, a non-apoptotic form of necrotic programmed cell death, named necroptosis, has been investigated in many biological and pathological processes. We then determine whether HIV-1-infected cells also undergo necroptosis. In this report, we demonstrate that HIV-1 not only induces apoptosis, but also mediates necroptosis in the infected primary CD4^+^ T lymphocytes and CD4^+^ T-cell lines. Necroptosis-dependent cytopathic effects are significantly increased in HIV-1-infected Jurkat cells that is lack of Fas-associated protein-containing death domain (FADD), indicating that necroptosis occurs as an alternative cell death mechanism in the absence of apoptosis. Unlike apoptosis, necroptosis mainly occurs in HIV-infected cells and spares bystander damage. Treatment with necrostatin-1(Nec-1), a RIP1 inhibitor that specifically blocks the necroptosis pathway, potently restrains HIV-1-induced cytopathic effect and interestingly, inhibits the formation of HIV-induced syncytia in CD4^+^ T-cell lines. This suggests that syncytia formation is mediated, at least partially, by necroptosis-related processes. Furthermore, we also found that the HIV-1 infection-augmented tumor necrosis factor-alpha (TNF-α) plays a key role in inducing necroptosis and HIV-1 Envelope and Tat proteins function as its co-factors. Taken together,necroptosis can function as an alternative cell death pathway in lieu of apoptosis during HIV-1 infection, thereby also contributing to HIV-1-induced cytopathic effects. Our results reveal that in addition to apoptosis, necroptosis also plays an important role in HIV-1-induced pathogenesis.

## Introduction

Necrosis used to be viewed as an accidental and unregulated process for cell death. However, accumulating evidence has suggested that necrosis, like apoptosis, can also occur in a coordinated and regulated manner, aptly termed ‘necroptosis’ [1]–[3]. Similar to the process of apoptosis activation, necroptosis is also triggered by tumor necrosis factor alpha (TNF-α), but leads to cell death independently of caspase-8 [4], [5]. Cellular morphology of necroptotic cells resembles that of necrotic cells, including loss of plasma membrane integrity, lack of nuclear fragmentation, mitochondrial dysfunction, and oxidative stress. It has been reported that the initiation of necroptosis by death receptors, such as tumor necrosis factor receptor 1 (TNFR1), requires the kinase activities of both receptor interacting protein 1 (RIP1) and 3 (RIP3) [6], [7]. Different experimental approaches have revealed the physical and functional interaction between RIP1 and RIP3 during necroptosis [8]–[10]. In particular, necrostatin-1 has been identified to specifically inhibit the kinase activity of RIP1, thereby undermining its interaction with RIP3 and antagonizing necroptosis, without affecting NF-κB [11]. From a system biology perspective, a set of 432 genes that specifically correlate to necroptotic murine cells has been identified, in which, 32 genes are regulators of RIP1 kinase and preferentially expressed in the innate immune and nervous systems [12]. Recent reports provided evidence that mixed lineage kinase domain like (MLKL) and phosphoglycerate mutase 5 (PGAM5) are integral parts of the necroptotic signaling machinery downstream of RIP1 and RIP3 activation and are the substrates of RIP3 [7], [13]–[15]. Furthermore, in order to identify putative RIP3 substrates, they screened a chemical library and identified a small molecule named necrosulfonamide (NSA), which inhibited necroptosis by covalently modifying MLKL [13], [15], [16].

Viral infection frequently induces cell death of which apoptosis is the major mechanism. However, a recent study observed RIP3-dependent necrotic cell death in response to murine cytomegalovirus (mCMV) infection in mice [17]. Additionally, RIP3^−/−^ mice are highly susceptible to vaccinia virus, indicating that necroptosis-related pathways could play a critical role in the antiviral process. Viruses also have mechanisms to antagonize the host cell death response. For example, the mCMV M36 protein inhibits death receptor-induced caspase-8 activation, while the viral M45 protein targets RIP3 and hinders TNF-induced NF-kB activation [18], [19]. By inhibiting apoptosis and necroptosis of infected cells, the virus buys itself time to replicate and proliferate within its host cells [20].

Human immunodeficiency virus type 1 (HIV-1) infection inevitably causes the exhaustion of CD4^+^ T lymphocytes largely due to apoptosis [21], [22]. HIV-1 encodes several apoptogetic proteins including envelop glycoprotein (Env), Vpr, and Tat, which cause direct viral cytotoxicity or signaling abnormalities [23]–[28]. However, the contribution of necroptosis to HIV-1-induced CD4^+^ T cell death remains unknown. In this study, we demonstrated that the infection of primary CD4^+^ T cells with either CXCR4-tropic HIV-1_NL4-3_ or CCR5-tropic HIV-1_YU2_ causes CD4^+^ T cell depletion by both necroptosis and apoptosis. We also showed that necroptosis mainly occurred in HIV-1-infected cells rather than in bystander cells. Hence, necroptosis is a novel mechanism that contributes to HIV-1-induced CD4^+^ T cell depletion.

## Materials and Methods

### Ethics statement

This research was approved by the Ethics Review Board of Sun Yat-Sen University. Written informed consent was provided by study participants and/or their legal guardians.

### Healthy donors

Healthy donors were comprised of a group of local volunteers, who were seronegative and had no reported history of chronic illness or intravenous drug use.

### Cell culture and reagents

The 293T and HT-29 cells were maintained in conditioned Dulbecco's modified Eagle's medium (DMEM) supplemented with 10% (vol/vol) fetal bovine serum (FBS), plus 100 μg/ml penicillin and streptomycin. The CD4^+^ T-cell lines such as H9, SupT1, wild-type and FADD-/- Jurkat (ATCC) were cultured in conditioned RPMI 1640 supplemented with 10% FBS and 100 μg/ml penicillin and streptomycin. Peripheral blood mononuclear cells (PBMCs) were obtained from HIV-1 seronegative donors, and isolated using Ficoll gradient centrifugation, followed by culturing in conditioned RPMI 1640 medium. The CD4^+^ T cells were then isolated by MACS microbead-negative sorting using human CD4^+^ T cell isolation kit (Miltenyi Biotec). The purity of CD4^+^ T cell fraction was higher than 95%. Subsequently, the primary CD4^+^ T cells were stimulated with phytohemagglutinin (PHA) (5 ng/ml) and interleukin-2 (IL-2) (10 ng/ml) for 48 hrs. The cells were then washed three times with phosphate-buffered saline (PBS), and cultured in the presence of IL-2 (10 ng/ml). Every 3–4 days, the culture was fed with half volume of fresh conditioned medium containing IL-2 (10 ng/ml). To specifically block necroptosis, 30 μM necrostatin-1 (nec-1) (Calbiochem), 2 μM necrosulfonamide (NSA) (Cellagen Technology) which was dissolved in DMSO, was added into the culture. For the pharmacological blockage of apoptosis, z-VAD-fmk (Sigma) was dissolved in DMSO and added into the culture at 20 μM.

### Construction of GFP and CCR5 expressing SupTI cell lines

The pBMN-I-GFP vector was obtained from Addgene. Recombinant MMLV viruses were generated by co-transfection of non-infectious MMLV vectors including pvPACK, pVSV-G, and pBMN-I-GFP constructs into 60% confluent HEK 293T cells (100 mm cell cultural dish) using Lipofectamine 2000 (Invitrogen) by following the manufacturer's protocol. Viral supernatant was collected at 48 hrs after transfection and used to infect SupT1 cells in the presence of 4 μg/ml polybrene. The infected (GFP-positive) cells were then sorted with a high-speed cell sorter (BD Influx). We replaced the *gfp* gene of the pBMN-I-GFP vector with human *ccr5* gene and used the same method to generate SupT1-CCR5 cells. Successfully-transduced cells were selected by flow-cytometry to sort the CCR5-positive cells using anti-CCR5 PE-labeled antibody or GFP-positive cells by GFP fluoresce (Ebioscience).

### HIV-1 production and infection

HIV-1 infectious clones, pNL4-3 (X4) and pYU2 (R5), including HIV-1 pseudovirus gene PNL4-3-ΔENV were amplified with HB101 competent cells (Promega). To generate viruses, 293T cells were transfected with 10 μg of infectious clones and Lipofectamine 2000 (Invitrogen) by following manufacturer's instructions. Culture supernatants were harvested at 48 hrs post-transfection and stored at −80°C. To normalize viral inputs, the amount of p24 was measured by HIV-1 p24 enzyme-linked immunosorbent assay (ELISA). The target cells (1×10^6^) were infected with the equivalent of 5 ng HIV-1 p24 in 1 ml for 3 hrs at 37°C. The virus-containing supernatants were then removed by washing 3 times with PBS. The cells were maintained in conditioned RPMI 1640 medium supplemented with IL-2 (10 ng/ml). HIV-1 replication was monitored by p24 detection.

### Flow cytometry

An ApoScreen Annexin-V apoptosis kit (Southern Biotech) was used for detecting apoptosis and necrosis. The manufacturer's protocol was followed with some modifications. Briefly, the cells were suspended in 10 μl cold 1× binding buffer in the range of 1×10^5^ to 1×10^6^ cells/ml, and mixed with 10 μl Annexin-V. After incubation at room temperature for 10 min in the dark, the mixtures were added with 380 μl of cold 1× binding buffer and 10 μl of 7-AAD. The stained cells were then analyzed by an EPICS XL apparatus (Beckman Counter) using a single laser emitting excitation light at a wavelength of 488 nm.

### Small Interfering RNA (siRNA) knockdown and receptor blocking experiments

The human TNFR1-specific siRNA and a control siRNA were purchased from Ribo, Inc. (Guangzhou, China). The primary CD4^+^T cells were transfected with 50 nM siRNA using Lipofectamine RNAimax (Invitrogen, Inc.). The cells were then treated with DMSO or Nec-1 after 6 hrs. The recombinant fusion protein YISAIPU, a recombinant fusion protein composed by human TNF-α receptor and Fc fragment of IgG (CP GuoJian-Pharm, Shanghai, China), was diluted in ddH_2_O and added into the culture for HIV-1-infected CD4^+^T cells (100 ng/ml). After 4 days, the cells were harvested and subjected to FACS analysis.

### Co-immunoprecipitation and Western blotting

In preparation for transfection, 1.5×10^6^ HeLa-CD4 cells were plated onto 60-millimeter (mm)-diameter cell culture plate and grown at 37°C in the conditioned Dulbecco's modified Eagle's medium (DMEM). The cells were then co-transfected with 3 μg pcDNA3.1-RIPK1-HA and 3 μg pcDNA3.1-RIPK3-FLAG. After 6 h, cells were infected with HIIV-1 viruses at 25 ng HIV-1 p24 equivalent and then treated with DMSO or Nec-1. Three days later, cells were collected and treated with lysis buffer [150 mM NaCl, 50 mM Tris-HCl (pH 7.5), 1 mM EDTA, 1% Triton X-100, and 0.5% NP-40]. Co-immunoprecipitation and Western blotting were then performed as previously described [29]. The anti-HA-Agarose (sigma) antibody, anti-HA antibody (mouse monoclonal, Covance), anti-FLAG antibody (rabbit polyclonal, MBL), anti-caspase3 antibody (rabbit polyclonal, CST), and anti-tublin antibody (mouse monoclonal, abcam) were used as primary antibodies.

### Mitochondrial membrane potential assay

Mitochondrial membrane potential assay kit with JC-1 was purchased from Keygen Biotech and performed exactly following manufacturer's instructions. Flow cytometry was performed for the final step.

### Quantization of Syncytia Formation

Syncytia formation was quantitated by phase contrast microscopy. Cells were cultured in 12-well plates and syncytia numbers counted per well. Fusion by 5 nuclei or more were calculated as single syncytia.

### Cell viability assay

Cell viability assay was the CellTiter-Glo Luminescent Cell Viability Assay Kit which was purchased from Progema Company. A CellTiter-Glo Assay was performed according to the manufacturer's instructions. Luminescence was recorded with a Progema plate reader.

### Statistics

Statistical analysis was done using t-tests with SPSS Statistics 17.0 and p values of <0.05 were considered significant.

## Results

### HIV-1-infection in primary CD4^+^ T cells and CD4^+^ T-cell lines induces necroptosis

Dual staining with fluorescent annexin-V and 7-AAD has been used to discriminate between apoptotic and necrotic cell death [30], [31]. When apoptosis is initiated, cells begin to display phosphatidylserine on the outer cellular membrane where it readily binds with annexin-V. On the other hand, 7-AAD is a chemical compound that intercalates double-stranded nucleic acids, but cannot readily pass through intact cellular membrane. Only upon permeabilization or disruption of the plasma membrane can 7-AAD enter the cells, bind DNA and fluoresce. Accordingly, apoptosis is characterized by annexin V-positive and 7-AAD-negative staining, while necrosis is characterized by annexin V-negative and 7-AAD-positive staining. Furthermore, necrostatin-1 (Nec-1), the RIP1 inhibitor specifically blocking necroptosis, was utilized to discriminate between necrosis and necroptosis [32].

**Figure 1:**
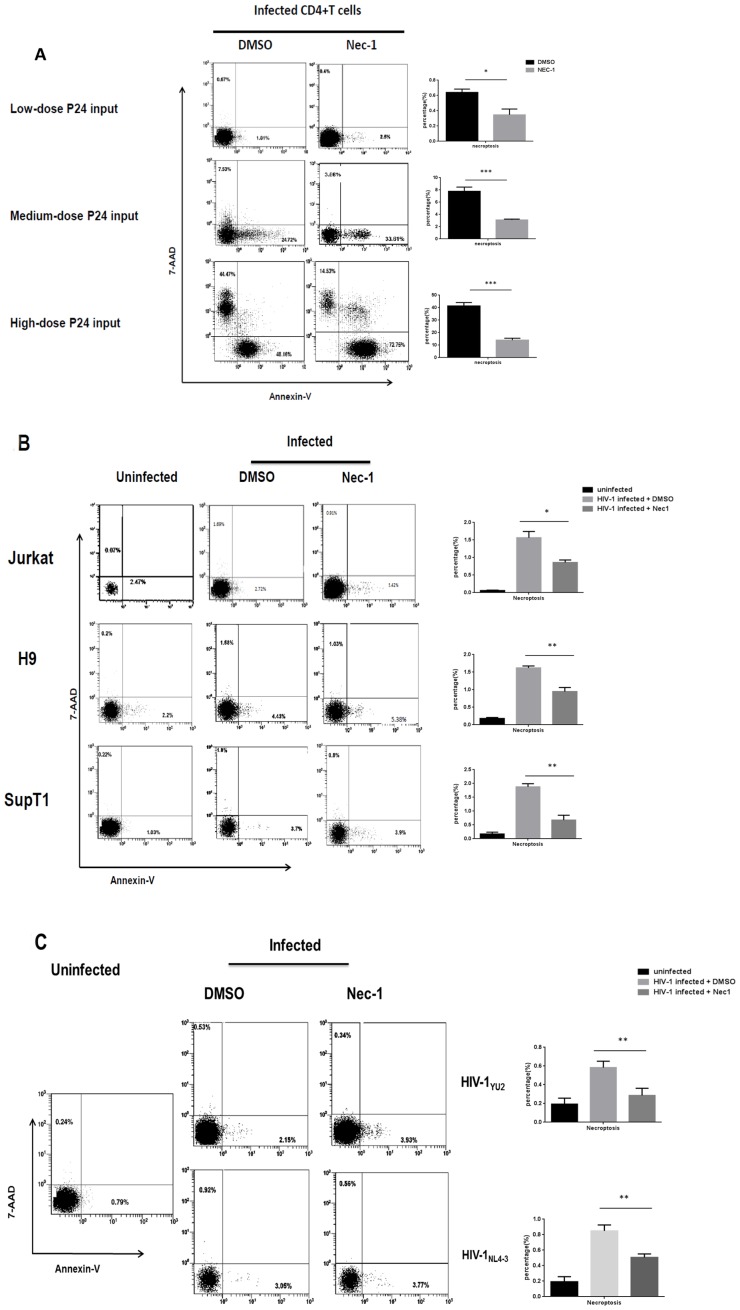
HIV-1 induces apoptosis and necroptosis in both primary CD4^+^ T cells and T-cell lines. **A**. The primary CD4^+^ T cells were infected with HIV-1_NL4-3_ at different doses with and without Nec-1 treatment. DMSO served as a negative control. At day 4 post-infection, cells were harvested and analyzed by flow cytometry and after, quantitation of necroptosis by histogram. (A high dose stands for 125 ng HIV-1 p24 per 10^6^ cells, a medium dose stands for 25 ng HIV-1 p24 per 10^6^ cells and a low dose stands for 5 ng HIV-1 p24 per 10^6^ cells.).*p<0.05, **p<0.01, ***p<0.001, n = 3. **B**. Three CD4^+^ T-cell lines, Jurkat, H9 and SupT1, were infected with HIV-1_NL4-3_ (5 ng HIV-1 p24 per 10^6^ cells), with and without the treatment of Nec-1. At day 4 post-infection, cells were harvested and analyzed by flow cytometry and after, quantitation of necroptosis by histogram. *p<0.05, **p<0.01, ***p<0.001, n = 3. **C**. Primary CD4^+^ T cells were infected with HIV-1_YU2_ and HIV-1_NL4-3_ (5 ng HIV-1 p24 per 10^6^ cells), with and without Nec-1 treatment. At day 4 post-infection, cells were harvested and analyzed by flow cytometry and after, quantitation of necroptosis by histogram. *p<0.05, **p<0.01, ***p<0.001, n = 3. Data in this figure is representative of at least 3 independent experiments.

To investigate whether necroptosis occurs during HIV-1 infection, primary CD4^+^ T lymphocytes were infected with CXCR4-tropic HIV-1_NL4-3_ in various doses (Fig. 1A and Fig. S1 in File S1, uninfected control). Necrosis and apoptosis were examined among infected and uninfected cells by staining with 7-AAD and annexin-V. A high dose (125 ng HIV-1 p24 per 10^6^ cells) HIV-1_NL4-3_ infection yielded 44.47% necrotic and 48.16% apoptotic cells, a medium dose (25 ng HIV-1 p24 per 10^6^ cells) HIV-1_NL4-3_ infection yielded 7.53% necrotic and 24.72% apoptotic cells, whereas a low dose (5 ng HIV-1 p24 per 10^6^ cells) infection yielded 0.67% necrotic and 1.81% apoptotic cells 4 days post-infection (Fig. 1A). Furthermore, we found that Nec-1 suppressed necrosis over 50% in HIV-1-infected CD4^+^ T cells, illustrating that the necrostic cell death was largely due to necroptosis (Fig. 1A). Meanwhile, we also found that HIV-1 infection induced necroptosis in CD4^+^ T-cell lines such as Jurkat, H9 and SupT1 (Fig. 1B).

**Figure 2:**
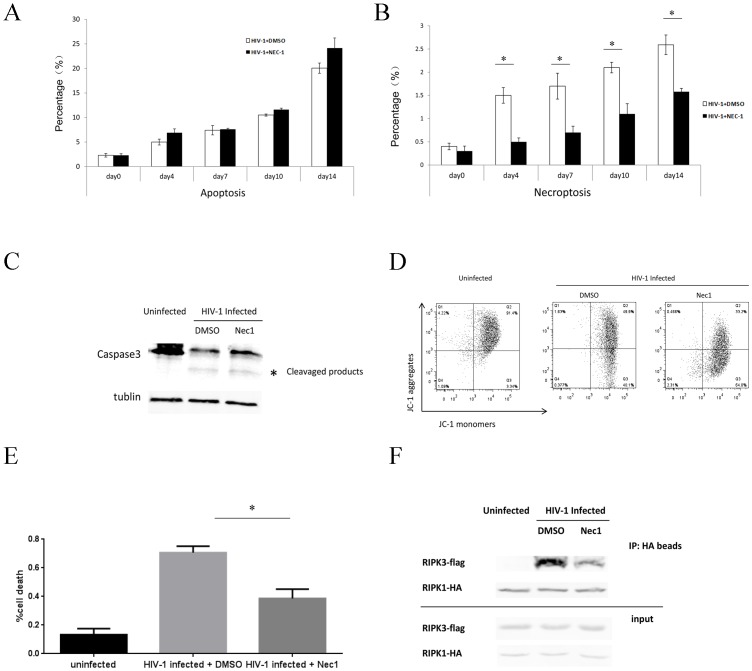
Time-course studies of necroptosis and apoptosis in HIV-1_NL4-3_-infected primary CD4^+^ T cells. A and B. Primary CD4^+^ T cells were infected with HIV-1_NL4-3_ (5 ng HIV-1 p24 per 10^6^ cells), and then with and without the treatment of Nec-1 or DMSO. The percentages of apoptosis (A) and necroptosis (B) were analyzed by flow cytometry at day 0, 4, 7, 10, and 14 post-infection. **C and D** The primary CD4^+^T cells were infected with HIV-1_NL4-3_ (25 ng HIV-1 p24 per 10^6^ cells) and then treated with or without Nec-1 treatment. After 4 days, cells were collected for (C) western blot to detect caspases-3 activation; or (D). mitochondrial membrane potential with JC-1 changes. The wave length 527 nm channel was used to detect JC-1 monomers and wave length 590 nm channel to detect JC-1 aggregates. **E**. Primary CD4^+^ T cells were infected with HIV-1_NL4-3_ (5 ng HIV-1 p24 per 10^6^ cells), with and without Nec-1 treatment. At day 4 post-infection, cells were harvested and analyzed by cell viability assay. **F**. Hela-CD4 cells were first co-transfected with 6 μg pcDNA3.1-RIPK1-HA and pcDNA3.1-RIPK3-FLAG and then infected with HIV-1_NL4-3_ (25 ng HIV-1 P24) 6 h p.t. After that, cells were treated with DMSO or Nec-1. Three days later, cells were collected for co-IP.

In accordance with the results of HIV-1_NL4-3_ infection, CCR5-tropic HIV-1_YU2_ strain was also capable of inducing Nec-1-sensitive necroptosis in primary CD4^+^ T cells (Fig. 1C). However, both necroptosis and apoptosis occurred at lower levels when induced by HIV-1_YU2_ compared to HIV-1_NL4-3_ infection. Low dose HIV-1_YU2_ infection induced 0.53% necrosis and 2.15% apoptosis, while the same dose of HIV-1_NL4-3_ infection induced 0.92% necrosis and 3.05% apoptosis. This could be attributed to HIV-1_NL4-3_ strain being more virulent and efficient in inducing cell death than HIV-1_YU2_ strain. Noticeably, the necrosis induced by all the HIV-1 strains was Nec-1-sensitive. Because higher dose (125 or 25 ng HIV-1 p24 per 10^6^ cells) HIV-1 infection can directly trigger massive cell death, we chose the lowest dose (5 ng HIV-1 p24 per 10^6^ cells) in the succeeding experiments. In a time-course analysis, we found that both apoptosis and necroptosis increased gradually, corresponding to increasing viral replication (Fig. 2A & 2B). It is notable that HIV-1-induced apoptosis was further supported by the activation of caspase-3 cleavage [33], [34] and the changed mitochondria potential feature [35], [36] (Fig. 2C & 2D). In Fig. 2C, we found that there is a cleavaged product of caspase-3 which indicated the activation of caspase-3 during HIV-1-induced apoptosis. In addition, we use JC-1, a method with the flow cytometry analysis for mitochondrial membrane potential in HIV-1 infected or uninfected cells. Of note, during apoptosis, JC-1 is capable of forming J-aggregates that are associated with a large shift in emission (527 nm to 590 nm) depending on the membrane potential. As a result, the color of the dye changes reversibly from green to greenish orange as the mitochondrial membrane becomes more polarized [37]. We found almost a half has the color changes during HIV-1 infection, indicating the apoptosis took place (Fig. 2D). Alternatively, to confirm the necroptosis during HIV-1 infection, cell viability assay, which has been used by other groups, was used and also confirmed the HIV-1-induced necroptosis [38], [39] (Fig. 2E). Further, because the specific combination of RIPK1 and RIPK3 lead to necroptosis and the nec-1 can inhibit their interaction, we have also performed RIPK1/RIPK3 co-IP to clarify that necroptosis occurs during HIV-1 infection (Fig. 2F). These results are in consistent with our hypothesis.

### Necroptosis correlates with syncytia formation in HIV-1-infected CD4^+^ T-cell lines

**Figure 3:**
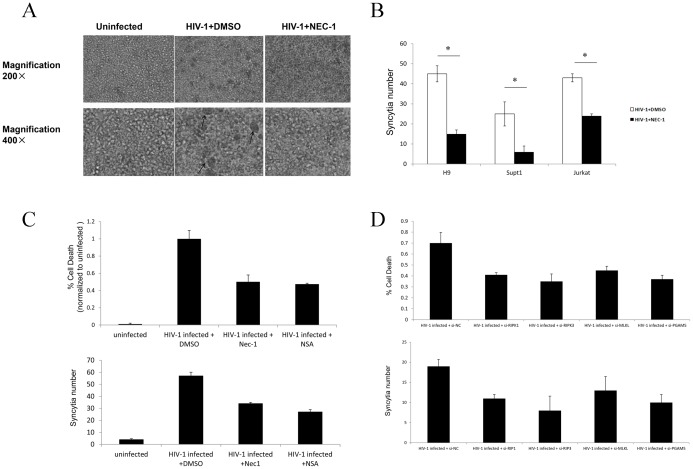
The correlation of necroptosis and syncytia formation in HIV-1_NL4-3_-infected T-cell lines. **A**. The morphological features of syncytia formation in HIV-1_NL4-3_ infected (5 ng HIV-1 p24 per 10^6^ cells) Jurkat cells at day 7 post-infection. The infected cells were treated with and without 30 μM Nec-1. The syncytia are indicated with the arrows. **B**. Quantitation of syncytia. *p<0.05, n = 3. **C**. Jurkat cells were infected with HIV-1_NL4-3_ infected (5 ng HIV-1 p24 per 10^6^ cells) and the infected cells were treated with DMSO, 30 μM Nec-1or 2 μM nsa. Quantitation of syncytia at day 7 post-infection. *p<0.05, n = 3. **D**. Jurkat cells were infected with HIV-1_NL4-3_ infected (5 ng HIV-1 p24 per 10^6^ cells) and then infected cells were transfected with 50 nM siRNAs and cultured in 24 well-plate. Quantitation of syncytia at day 7 post-infection. *p<0.05, n = 3.

HIV-1 enters target cells by direct fusion of the viral and host cell membranes [40]. The fusion reaction is initiated by the interaction between gp120/gp41 and CD4 on the surface of target cells. By a similar mechanism, infected cells expressing Env can fuse with CD4^+^ cells, leading to the formation of syncytia [41]–[44]. Syncytia formation is a major death pathway for CD4^+^ T-cell lines infected with HIV-1 and has long been considered as a marker of HIV-1 infection in these cell lines. Addition of Nec-1 to HIV-1-infected H9, SupT1, and Jurkat cell lines blocked necroptosis and dramatically reduced syncytia formation (Fig. 3A, upper and lower panel). There was more significant reduction of the larger syncytia (fusion of ≥ 5 nuclei) compared to the smaller ones (fusion of < 5 nuclei) upon Nec-1 treatment. Necroptosis inhibition with Nec-1 occurred in conjunction with reduced syncytia and was detected by FACS analysis (Fig. 3B and Fig.S2 in File S1).

To further demonstrate the correlation of necroptosis and syncytia formation, we examined the effects of other specific inhibitors for necroptosis pathway. Recently, another small molecule named necrosulfonamide (NSA) has been identified by Wang and his colleagues to block necroptosis [15]. We found that both Nec-1 and NSA blocked necroptosis in a dose-dependent way by cell viability assay (Fig. S3 in File S1). Meanwhile, NSA also could down-regulated the formation of syncytia (Fig. 3C). As several genes including RIPK1, RIPK3, MLKL and PGAM5 play important roles in necroptosis [8], [14], [15], [45], we designed their corresponding siRNAs to inhibit their expression. All the siRNAs, which effectively decreased their target mRNAs respectively (Fig. S4 in File S1), significantly inhibited syncytia formation and reduced necroptosis (Fig. 3D). These data further supported the hypothesis that syncytia could be correlated with the signaling pathway of necroptosis.

### The inhibition of apoptosis enhances necroptosis in HIV-1-infected cells

**Figure 4:**
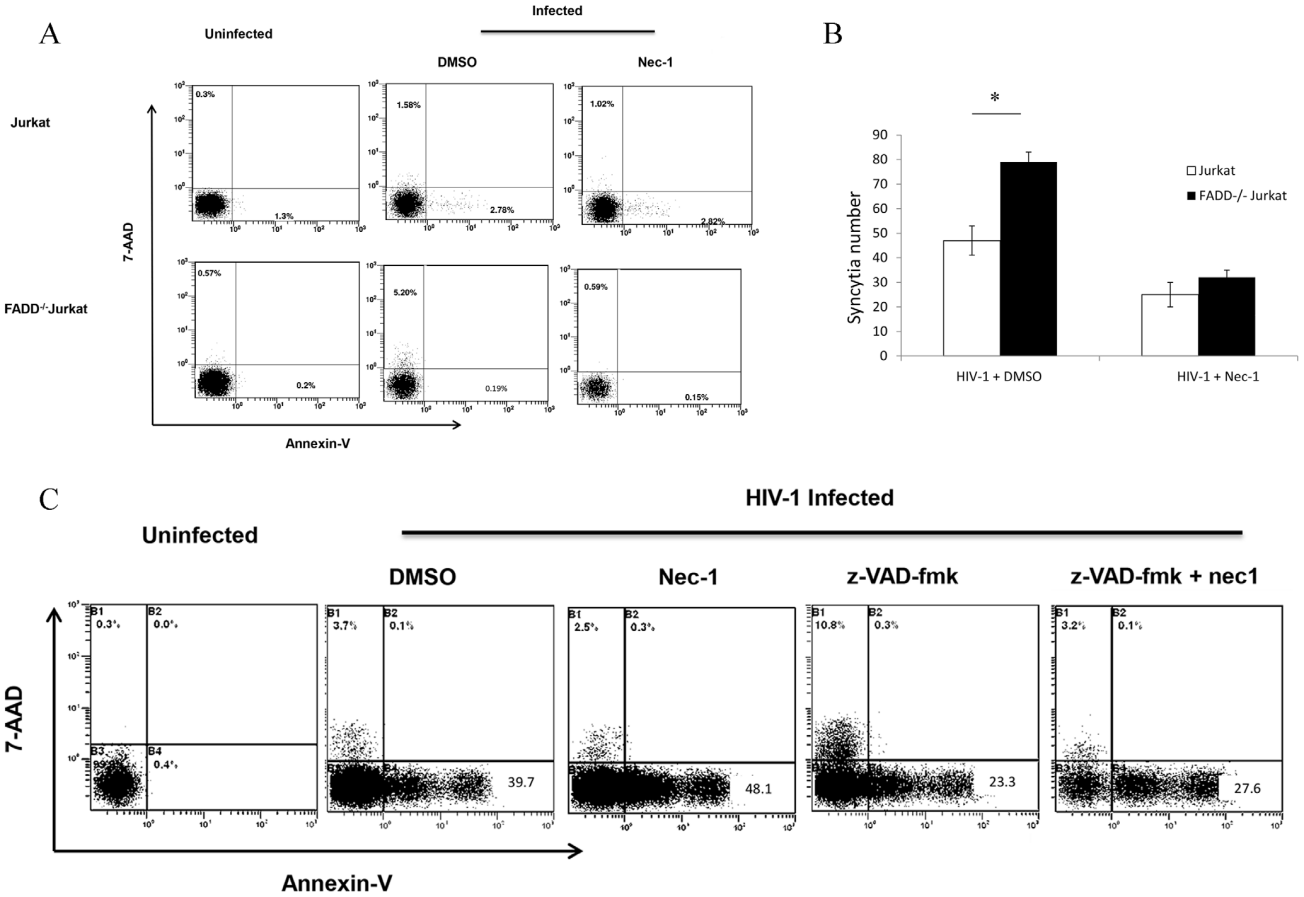
Enhancement of necroptosis in HIV-infected FADD^−/−^ Jurkat cells. **A**. Wild-type and FADD^−/−^ Jurkat cells were infected with HIV-1_NL4-3_ (5 ng HIV-1 p24 per 10^6^ cells), with and without 30 μM Nec-1 treatment. After 4 days, cells were harvested and analyzed by flow cytometry. **B**. Quantitative analysis of syncytia. *p<0.05, n = 3. **C**. Wild-type Jurkat cells were infected with HIV-1_NL4-3_ (5 ng HIV-1 p24 per 10^6^ cells) and then treated with DMSO, Nec-1, z-VAD-fmk, or z-VAD-fmk plus Nec-1 respectively. After 4 days, cells were harvested and analyzed by flow cytometry.

To further investigate the possible relation between necroptosis and apoptosis for HIV-1-induced cell death, we infected a FADD-/- Jurkat cell line with HIV-1_NL4-3_. The Fas pathway, with the corresponding downstream caspases, is completely disengaged in these FADD-/- cells [46]. We found that necroptosis increased from 1.58% in wildtype to 5.20% in FADD-/- HIV-1-infected Jurkat cells (Fig. 4A), albeit the kinetics of viral infection was the same in both cell lines (Fig. S5 in File S1). Of note, low level residual apoptosis still occurred in FADD-/- Jurkat cells (Fig. 4A). Interestingly, compared with wild type cells, the formation of syncytia also increased in FADD-/- Jurkat cells post-infection (Fig. 4B). Alternatively, when we used a caspase inhibitor z-VAD-fmk [47] to block caspase activation a similar result was found, which is in consistent with the result after genetic disruption of FADD (Fig. 4C). Again, syncytia formation was inhibited by Nec-1 in both cell lines, further supporting the correlation between necroptosis and syncytia formation.

### Necroptosis mainly takes place in infected cells

**Figure 5:**
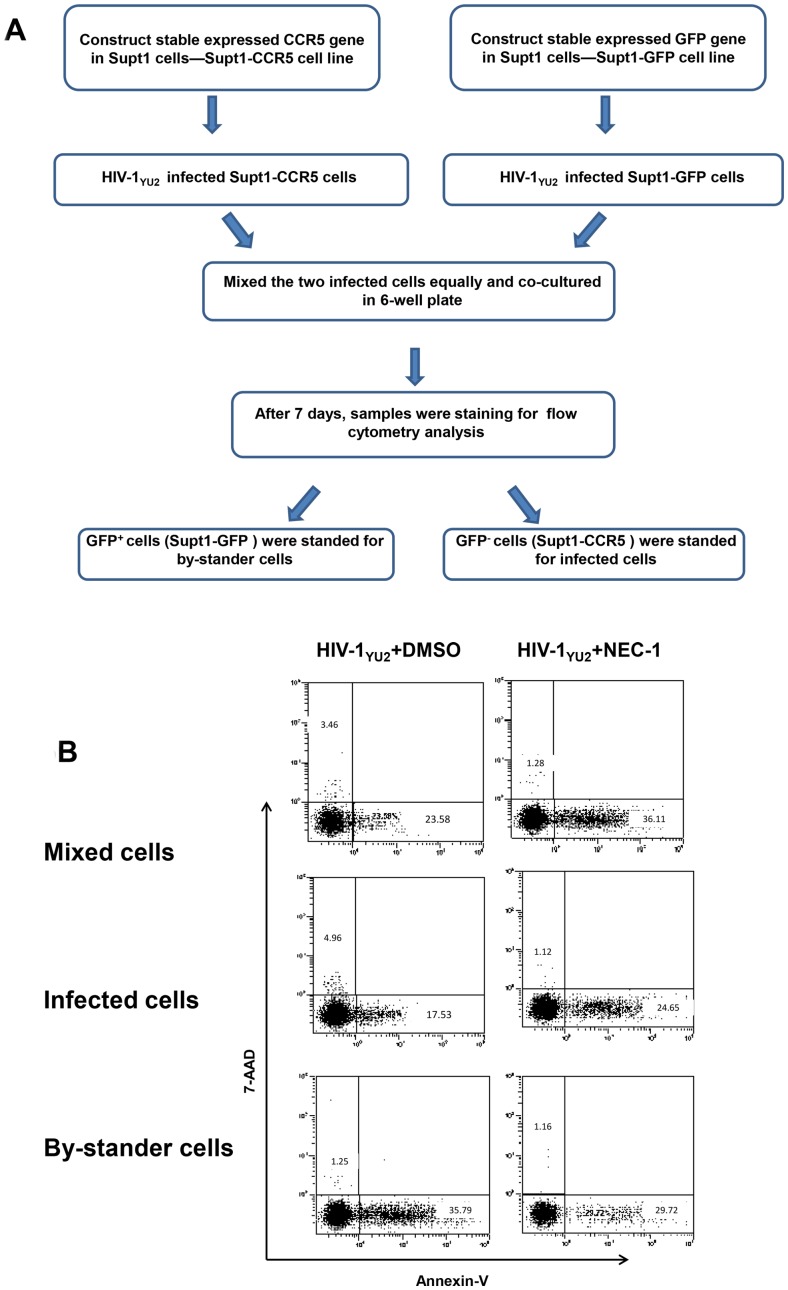
Necroptosis mainly takes place in HIV-1-infected cells. **A**. Schematic presentation of methodology. **B**. SupT1-GFP and SupT1-CCR5 cells were infected with HIV-1_YU2_ (5 ng HIV-1 p24 per 10^6^ cells), with and without the 30 μM Nec-1 treatment. After 4 days, cells were harvested and analyzed by flow cytometry. Data in this figure is representative of at least 3 independent experiments.

As the apoptosis induced by HIV-1 infection mainly occurs in bystander cells, it is interesting to determine whether necroptosis is a direct or bystander effect of HIV-1 infection. It is known that SupT1 cells are CXCR4-expressing cells and susceptible to infection by X4-tropic, but not by R5-tropic HIV-1 strains [48]. Through retroviral transduction and subsequent marker selection via FACS, we constructed two modified cell lines: SupT1-GFP and SupT1-CCR5, which stably expressed GFP and CCR5 proteins, respectively. Through a co-culture experiment using both kinds of modified SupT1 cells, we found that the HIV-1_YU2_ infection induced 3.46% necrosis and 23.58% apoptosis in the mixture of the two kinds of SupT-1 cells. Specifically, HIV-1_YU2_ induced 4.96% necrosis in directly infected SupT1-CCR5 cells and 1.25% in bystander SupT1-GFP cells. The Nec-1 treatment significantly inhibited the necrosis in directly infected SupT1-CCR5 cells but almost not in bystander SupT1-GFP cells (Fig. 5B and Fig. S6 in File S1). Alternatively, the induction of apoptosis in bystander T cells has been repeatedly implicated as a mechanism contributing to the T-cell depletion in *in vivo* HIV-1 infection [28], [49]. In line with these reports, we observed that apoptosis mainly occurred in bystander cells at 35.79%, while the apoptosis percentage of infected cells in co-culture was 17.53% (Fig. 5B). Taken together, our data indicate that HIV-1-induced necroptosis occurs in infected rather than uninfected cells, which is distinct from what happens in HIV-1-induced apoptosis.

### The increased TNF-α production and HIV-1 Envelope and Tat proteins are involved in necroptosis

**Figure 6:**
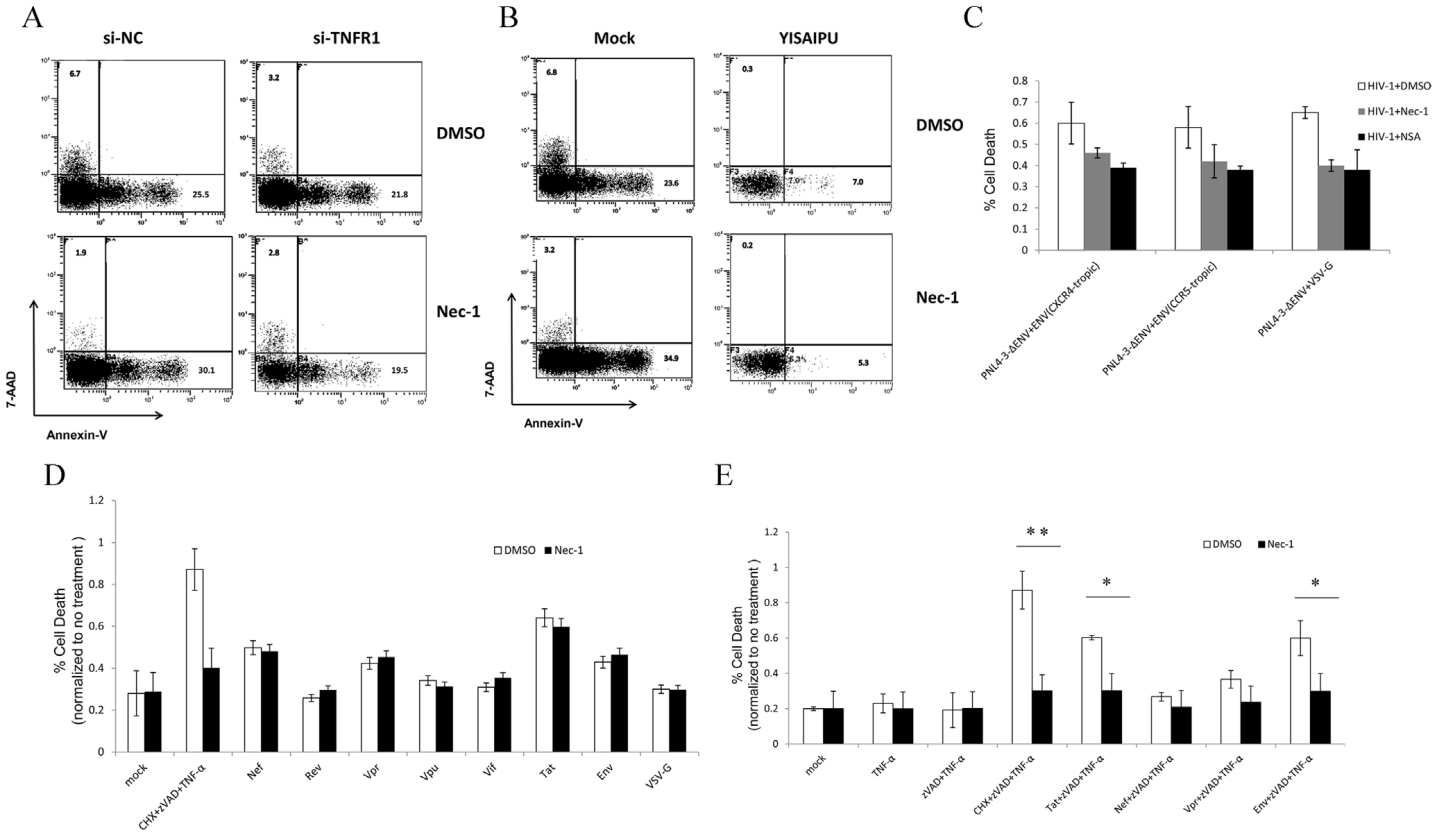
The increased TNF-α production and HIV-1 Envelope and Tat proteins are involved in necroptosis. A.Primary CD4^+^T cells were first infected with HIV-1_NL4-3_ (5 ng HIV-1 p24 per 10^6^ cells), and then cells were transfected with 50 nM siRNA at 4 hrs post-infection. After that, DMSO or Nec-1 was added into cells. At day 4 post-infetion, cells were harvested and analyzed by flow cytometry. B?Primary CD4^+^T cells were first infected with HIV-1_NL4-3_ (5 ng HIV-1 p24 per 10^6^ cells), and then cells were treated with 100 ng/ml YISAIPU at 4 hrs post-infection. After that, DMSO or Nec-1 was added into cells. At day 4 post-infetion, cells were harvested and analyzed by flow cytometry. **C**. The primary CD4^+^ T cells were infected with three types of HIV-1 pseudoviruses which were packaged by pNL4-3-env plus CXCR4-tropic, CCR5-tropic, or VSV-G envelope gene. After infection, cells were treated with DMSO, 30 μM Nec-1 or 2 μM NSA. At day 4 post-infection, cells were harvested and analyzed by cell viability assay. **D**. HT-29 cells were transfected with plasmids expressing different HIV-1 proteins and also treated with or without Nec-1. After 3 days, cells were harvested and analyzed by cell viability assay. **E**. HT-29 cells were transfected with plasmids expressing HIV-1 Envelope, Tat, Nef, Vpr proteins and after 6 hrs, cells were treated with TNF-α plus ZVAD. Then, DMSO or Nec-1 was added into cells. After 3 days, cells were harvested and analyzed by cell viability assay. *p<0.05, **p<0.01, n = 3.

As TNF-α plays an important role during necroptosis and its generation from T-lymphocytes significantly increased during HIV-1 infection [50] (Fig. S7 in File S1), we sought to determine whether the TNF-α is also a key factor for HIV-1-induced necroptosis. After depletion of TNFR1 with siRNA, we found that HIV-1-induced necroptosis was inhibited from 6.7% to 3.2% (Fig. 6A). Additionally,YISAIPU, a recombinant fusion protein composed by human TNF-α receptor and Fc fragment of IgG, was added into the cell culture to competitively inhibit the interaction between TNF-α and TNFR [51]. We found the necroptosis was also significantly inhibited from 6.8% to 0.3% (Fig. 6B).

To investigate the role of viral proteins in HIV-1-induced necroptosis, we first compared the effect of different recombinant HIV-1 viruses pseudotyped with CXCR4-tropic envelope, CCR5-tropic envelope, or VSV-G (an envelope of vesicular stomatitis virus) on necroptosis. Our data showed that neither CXCR4-tropic envelope nor CCR5-tropic envelope mediated the necroptosis during the viral entrance step, as all of three recombinant HIV-1 viruses leaded to necroptosis cell death at almost same level (Fig. 6C).

Because the CD4^+^ T-cell lines are suspension cell lines and it is difficult to transfect plasmids into these cells, we utilized HT-29, an adherent cell line which has routinely been used for necroptosis studies [10], [39], to further examine the possible effect of HIV-1 proteins [15]. Initially, we transfected constructs expressing various viral proteins into this cell line respectively. Although some HIV-1 proteins such as Tat, Nef, or Vpr alone induce cell death, it is unlikely that the cell death is due to necroptosis as Nec-1 cannot rescue the cell death induced by these proteins (Fig. 6D). It has been shown that necroptosis induced in HT-29 routinely require the combination of TNF-α, cycloheximide (CHX), and Z-VAD-FMK. Among them, CHX is a pan-inducer of cell death and ZVAD-FMK is a pan-caspase inhibitor. We then tried to use several HIV-1 proteins, which had been shown to be associated with cytopathic effect of HIV-1, to replace of CHX. When HIV-1 Envelope, Tat, Nef, Vpr proteins were combined with TNF-α in the presence of ZVAD, we found that Envelope or Tat protein can effectively replace CHX and induced necroptosis in HT-29 cells (Fig. 6E).

## Discussion

The CD4^+^ T cell depletion observed during HIV-1 infection is one of the primary mechanisms for HIV-1 pathogenesis. In this report,we demonstrated that necroptosis was a novel pathway accounting for the death of HIV-1-infected primary CD4^+^ T lymphocytes and other CD4^+^ T-cell lines. The proportion of necroptosis was increased with higher viral input and the contribution of necroptosis to HIV-1-induced cell death was not as considerable as that of apoptosis. Furthermore, we found that necroptosis mainly occurred in the infected cells rather than in the uninfected bystander cells. In contrast, our data showed that most apoptosis occurred in the uninfected bystander cells, which is consistent with other reports [28], [49].

Interactions between HIV-1-infected and neighboring uninfected cells often lead to cell fusion and the formation of multi-nucleated syncytial cells, which have been observed both in vitro and in vivo in different organs including the brain [52]–[54]. The syncytia induced by specific virus strains in T cell lines are frequently correlated with the final phase of AIDS progression [42], [55], [56]. The molecular mechanism of syncytia formation remains elusive. It has been proposed that the HIV-1 envelope protein could trigger and facilitate cell-cell fusion [42], [57]. Indeed, many enveloped viruses have a fusion peptide which enables the fusion of viral and host membranes to allow the entrance of infectious genomic materials into the cytoplasm. During viral replication, the expression of envelope protein on the host cellular membrane leads to its fusion with neighboring cells and syncytia formation. However, the details of this process remain unclear. Our data show a strong correlation between syncytia formation and necroptosis. Specifically, syncytia formation is significantly decreased upon Nec-1, NSA, or specific siRNA inhibition of necroptosis in HIV-1-infected cells. At this point, we cannot conclude whether necroptosis is a cause or a consequence of syncytia formation. It would be worthwhile to further investigate the molecular mechanisms underlying this phenomenon. As syncytia formation is frequently observed during the infection of many enveloped viruses, it would also be interesting to examine if a similar correlation exists between necroptosis and syncytia formation in other enveloped virus infections.

As previously mentioned, FADD-/- Jurkat cells are incapable of undergoing apoptosis due to the disruption of the extrinsic apoptotic pathway [46]. Indeed, HIV-1-infection-induced apoptosis in FADD-/- Jurkat cells dramatically decreased, while necroptosis significantly increased, compared to that in wild-type Jurkat cells. In addition of increased necroptosis, syncytia formation also increased in these cells. Again, nec-1 significantly interrupted necroptosis and syncytia formation both in HIV-1-infected wild-type and FADD-/- Jurkat cells. These results demonstrate that necroptosis may act as an alternative and compensatory cell death pathway when apoptosis cannot effectively mediate HIV-1 induced cell death. Conversely, when necroptosis is inhibited, the number of annexin-V-positive apoptotic cells increases. However, more evidence is needed to confirm that necroptosis and apoptosis are certainly the compensatory cell death mechanisms during HIV-1 infection.

We have preliminarily examined the possible viral factor(s) which could directly participate in HIV-1-induced necroptosis. As HIV-1 envelope proteins participate in the HIV-1-induced apoptosis [41], [42], it is important to determine whether envelope proteins also play a role in necroptosis. When we infected primary CD4^+^ T cells with HIV-1 pseudoviruses packaged from pNL4-3-Δenv which lack the envelop genes plus CXCR4-tropic, CCR5-tropic HIV-1 envelope, or VSV-G envelope, all of these three kinds of pseudoviruses still induced necroptosis at the similar level (Fig. 6C). These data show that viral envelope protein at the entrance event is not directly involved in HIV-1-induced necroptosis. Thus, it is unlikely that the signal transduction mediated by CD4 or CCR5/CXCXR4 activation is involved in triggering necroptosis.

TNF-α plays an important role in progression to AIDS for HIV-1-infected patients. High levels of TNF-α has been found in the in supernatants of PBMC from HIV-1 patients [50], [58], [59]. We also confirmed that TNF-α is significantly increased during HIV-1 infection in primary CD4+ T-lymphocytes (Fig. S7 in File S1). Interestingly, it has been reported that HIV-1 Tat protein induces the release of TNF-α in different types of cells [60], [61]. In this report, we found that TNF-α induced during HIV-1 infection play a key role in HIV-1-induced necroptosis. This result is consistent with a recent report that type I interferon(IFN-I) induces necroptosis in macrophage during bacterial infection via induction of TNF-α [62]. Furthermore, we also found that Envelope protein or Tat can function as a co-factor for TNF-α to induce necroptosis.

In summary, our results have clearly indicated that necroptosis occurs during HIV-1 infection. It would be interesting to continue exploring other key factors that could activate or regulate apoptosis and necroptosis pathways during HIV-1 infection. Since necroptosis research is still an emerging field, HIV-1-induced necroptosis could serve as an ideal model for further studying the molecular mechanisms of this newly defined non-apoptotic programmed cell death pathway.

## Supporting Information

File S1 **Supporting Figures.** Figure S1 - Uninfected cells grow with a low percentage of apoptosis and necrosis. Various cells were cultured in conditioned RPMI 1640 medium. After 4 days, cells were harvested and analyzed by flow cytometry together with HIV-1-infected cells. Data in this figure is representative of at least 3 independent experiments. Figure S2 - Nec-1 has no effect on uninfected cells. The primary CD4^+^T cells were cultured in conditioned RPMI 1640 medium and cells were treated with DMSO or Nec-1. After 4 days, cells were harvested and analyzed by flow cytometry. Data in this figure is representative of at least 3 independent experiments. Figure S3 - Nec-1 or NSA inhibited necroptosis in a dose-dependent way. The primary CD4^+^T cells were infected with HIV-1_NL4-3_ (5 ng HIV-1 p24 per 10^6^ cells), with different concentrations of Nec-1 or NSA treatment. After 4 days, cells were harvested and analyzed by cell viability assay. Figure S4 - Detection the knockdown efficacy of target siRNAs. The primary CD4^+^T cells were first infected with HIV-1_NL4-3_ (5 ng HIV-1 p24 per 10^6^ cells). After washing twice with PBS, infected cells were transfected with 30 nM siRNA and cultured in 24 well-plate. After 4 days, cells were harvested to extract RNA and the mNRA expression level of target genes were detected by RT-PCR. Figure S5 - The kinetics of viral infection is similar in both Jurkat cell lines. The wild-type and FADD-/-Jurkat cells were infected with HIV-1_NL4-3_ (5 ng HIV-1 p24 per 10^6^ cells). After 4 and 7 days, cell supernatant was harvested and analyzed by p24 ELISA assay. *p<0.05, n = 3. Figure S6 - Viral infection and cytopathic effect in separately infected cell lines. A. SupT1-GFP and SupT1-CCR5 cells were respectively infected with HIV-1_YU2_ (5 ng HIV-1 p24 per 10^6^ cells) and then cultured in conditioned RPMI 1640 medium. After 4 or 7 days, the uninfected and infected cells were both harvested and analyzed by flow cytometry. B. Cell supernatant was harvested and analyzed by p24 ELISA assay. *p<0.05, n = 3. Figure S7 – TNF-αwas significantly increased during HIV-1 infection. The primary CD4^+^T cells were infected with HIV-1_NL4-3_ (5 ng HIV-1 p24 per 10^6^ cells). After 4 days, cell supernatant were collected and analyzed by TNF-α ELISA kit. *p<0.05, n = 3.
(RAR)
